# The Evolution of Medical Student Competencies and Attitudes in Digital Health Between 2016 and 2022: Comparative Cross-Sectional Study

**DOI:** 10.2196/67423

**Published:** 2025-07-31

**Authors:** Paula Veikkolainen, Timo Tuovinen, Petri Kulmala, Erika Jarva, Jonna Juntunen, Anna-Maria Tuomikoski, Merja Männistö, Teemu Pihlajasalo, Jarmo Reponen

**Affiliations:** 1 FinnTelemedicum, Research Unit of Health Sciences and Technology, Faculty of Medicine, University of Oulu, Aapistie 5a, Oulu, 90220, Finland, 358 29 4480000; 2 Medical Research Center, the Wellbeing Services County of North Ostrobothnia and the University of Oulu, Oulu, Finland; 3 Education Development and Service Unit, Faculty of Medicine, University of Oulu, Oulu, Finland; 4 Research Unit of Health Sciences and Technology, Faculty of Medicine, University of Oulu, Oulu, Finland; 5 Oulu University Hospital, Wellbeing Services County of North Ostrobothnia, Oulu, Finland; 6 School of Health and Social Studies, JAMK University of Applied Sciences, Jyväskylä, Finland; 7 Mehiläinen Vaasa, Mehiläinen Health Services, Vaasa, Finland

**Keywords:** digital health, eHealth, telemedicine, medical informatics, professional competence, medical education, digitalization, digital health, digital, technology, medical student, cross-sectional study, health care systems, health care, health care professional, health care information system, survey, questionnaire, healthcare digitalization, digital competence, innovation

## Abstract

**Background:**

Modern health care systems worldwide are facing challenges, and digitalization is viewed as a way to strengthen health care globally. As health care systems become more digital, it is essential to assess health care professionals’ competencies and skills to ensure they can adapt to new practices, policies, and workflows effectively.

**Objective:**

The aim of this study was to analyze how the attitudes, skills, and knowledge of medical students concerning digital health have shifted from 2016 to 2022 in connection with the development of the national health care information system architecture using the clinical adoption meta-model framework.

**Methods:**

The study population consisted of 5th-year medical students from the University of Oulu in Finland during 2016, 2021, and 2022. A survey questionnaire was administered comprising 7 background questions and 16 statements rated on a 5-point Likert scale assessing students’ attitudes toward digital health and their self-perceived digital capabilities. The results were recategorized into a dichotomous scale. The statistical analysis used Pearson *χ*^2^ test. The Benjamini-Hochberg procedure was used for multiple variable correction.

**Results:**

The study included 215 medical students (n=45 in 2016, n=106 in 2021, and n=64 in 2022) with an overall response rate of 53% (43% in 2016, 74% in 2021, and 42% in 2022). Throughout 2016, 2021, and 2022, medical students maintained positive attitudes toward using patient-generated information and digital applications in patient care. Their self-perceived knowledge of the national patient portal significantly improved, with agreement increasing by 35 percentage points from 2016 to 2021 (*P*<.001) and this trend continued in 2022 (*P*<.001). However, their perceived skills in using electronic medical records did not show significant changes. Additionally, students’ perceptions of the impact of digitalization on health promotion improved markedly from 2016 to 2021 (with agreement rising from 53% to 78%, *P*=.002) but declined notably again by 2022.

**Conclusions:**

Medical students’ attitudes and self-perceived competencies have shifted over the years, potentially influenced by the national health information system architecture developments. However, these positive changes have not followed a completely linear trajectory. To address these gaps, educational institutions and policy makers should integrate more digital health topics into medical curricula and provide practical experience with digital technologies to keep professionals up-to-date with the evolving health care environment.

## Introduction

Modern health care systems around the world are facing challenges due to the aging population, increasing prevalence of chronic diseases, and other lifestyle-associated conditions [1,2]. At the same time, countries are grappling with shortages of the health care workforce, especially in remote and rural areas [3]. Different exposures to health risks create health inequalities between individuals with higher and lower education and income levels [4,5]. These factors put pressure on health care systems to shift their focus toward promoting health and preventing diseases through patient engagement and self-management.

According to the World Health Organization (WHO), the term “eHealth” focuses on using information and communication technologies in health care, while “digital health” serves as a broader umbrella term that also encompasses advanced computer sciences such as artificial intelligence. Furthermore, the term “mHealth,” a subset of eHealth, is defined as “the use of mobile wireless technologies for health” [2]. Regardless, digital transformation is seen as an essential component and enabler to enhance the quality, accessibility, and affordability of health services [2,6]. In 2021, the WHO published the Global Strategy on Digital Health 2020‐2025, seeking to assist nations in strengthening their health care systems through digitalization [7]. The European Union (EU) has named digital solutions as one of the key enablers to deliver health and care services more effectively to patients [8,9]. The COVID-19 pandemic, starting in 2020, and the resulting lockdowns accelerated the technological leap by forcing health care institutions worldwide to swiftly develop and implement digital strategies [10-13].

In 2022, Finland was ranked as the top country in the annual Digital Economy and Society Index report, which monitors the digital progress of the European Union Member States [14]. Accordingly, Finland has a long history of enforcing digitalization in health care [15,16]. One example of this development is the introduction and implementation of the Finnish nationwide, centralized shared electronic data system service, called the Kanta Services, which comprises several service entities such as an electronic patient portal, prescription database, and patient data repository. The implementation of these services has taken place in several stages throughout the 2010s [17-19].

In Finland, the key competence requirements for a graduating doctor have been established at a national level [20]. These requirements are based on international literature, including the UK’s Generic Professional Capabilities framework and the International Association for Medical Education guidelines [21-23]. The basic education for medical professionals in Finland consists of 2 years of pre-clinical studies followed by 4 years of clinical training. In the EU, the profession of medical doctor is recognized as a qualification in all member states on the basis of harmonized minimum training requirements [24].

As health care systems become more digital, it’s essential to assess health care professionals’ competencies and skills to ensure they can adapt to new practices, policies, and workflows effectively [25]. According to current literature, medical students globally have positive attitudes toward learning about digital health and consider the introduction of digital health topics into the medical curricula to be important [26-34]. However, more reserved perceptions toward digital health have also been reported among students [35].

Many nations have made efforts to develop and implement national digital health strategies, including initiatives to incorporate digital health education at local and national levels [36-40]. Furthermore, the European Medical Students’ Association has implemented policies emphasizing the inclusion of digital health in medical education curriculum to ensure future doctors in Europe possess crucial digital skills [41], and the recent Digital Health Competencies in Medical Education framework outlines the essential digital competencies for medical education on a global level [42]. Efforts have been made to modernize basic medical education in Finland as well; an example of this is the national MEDigi project (2018‐2021) funded by the Finnish Ministry of Education and Culture [43]. In addition to digitizing the teaching of medicine and dentistry, the project aimed to ensure a high level of competence in the use of digital health care tools among medical students and to establish national eHealth competence themes [44].

In our previous study, we aimed to compare the attitudes of medical and nursing students toward digital health. Based on the study results, the differences between the 2 student groups were small, and overall, the students’ attitudes toward digital health were positive [33]. Now, we seek to deepen our understanding of the changes in medical students’ attitudes, skills, and knowledge regarding digital health in relation to the underlying development of the national health care information system architecture. For this purpose, we used the clinical adoption meta-model (CAMM) framework. This framework is developed to describe the health information system adoption over time, and it incorporates 4 dimensions: availability, use, behavior changes, and outcome changes [45]. In this study, the aim is to focus on the third dimension of the model, namely, to describe the behavior changes (attitudes and competencies) of medical students in connection with the development of the national health care information system architecture over time.

## Methods

### Ethical Considerations

The research was conducted in accordance with the instructions of the Finnish Advisory Board on Research Integrity [46], and in compliance with EU data protection regulations [47] as well as the established research practices of the University of Oulu and the Faculty of Medicine. Therefore, no approval from the ethics committee was required. Full consideration was given to matters related to data protection in accordance with the ethical principles applicable to research subjects. Participation in the study was voluntary, and students were asked for their consent to collect and use data for the purpose of the study. The students were informed of the purpose of the study and their right to withdraw and prohibit the use of their data at any time. No incentives were offered for participation.

### Study Design

The study population for this comparative cross-sectional study consisted of 5th-year medical students at the University of Oulu who participated in a compulsory 1-day digital health course held in spring 2016, 2021, and 2022. The aim of this 1-day course was to provide essential knowledge on digital health and its applications from the perspective of health care professionals. There were 105, 144, and 154 medical students who enrolled in the courses in 2016, 2021, and 2022, respectively. The students were invited to participate in the study via email in 2016, as well as through the course’s Moodle environment in 2021 and 2022.

This study adheres to the EQUATOR CROSS (A Consensus-Based Checklist for Reporting of Survey Studies) guidelines for survey research [48]. The checklist was used to ensure comprehensive reporting of study design, data collection, analysis, and interpretation.

### Study Questionnaire

After completing the digital health course, a web-based survey was conducted on the students’ perceptions of digital health using a Webropol survey tool. The survey was compiled in 2016, and it was developed based on literature and expert reviews [49]. The survey was piloted prior to its use. The pilot group consisted of 2 fifth-year medical students and 2 medical teachers, both of whom had backgrounds in teaching digital health.

The Finnish-language survey questionnaire consisted of 16 statements (Q1-Q16) measured on a 5-point Likert scale (“Fully disagree” to “Fully agree”), surveying students’ attitudes to digital health and their self-perceived digital competencies. The survey used in 2021 and 2022 was adapted from the 2016 survey by changing the term “medical doctor” to “health care professional,” as nursing students also participated in the 1-day course in 2021 and 2022. An English translation of the latter questionnaire is presented in Multimedia Appendix 1. The statements were related to 5 themes concerning digital health: (1) the usage of patient-generated information and the role of digital applications in patient care; (2) health information systems; (3) digitalization of the working environment; (4) the changing role of patients and professionals; and (5) the culture of experimentation and readiness to participate in innovation activities.

### Data Analysis

The 5-point Likert-scale responses “Fully agree” and “Somewhat agree” were combined to form the category “Agree.” Similarly, the responses “Fully disagree,” “Somewhat disagree,” and “Neither agree or disagree” were combined to form the category “Disagree” using Excel (Microsoft Corp.).

The data analysis was conducted using a Pearson *χ*^2^ test to examine relationships between the medical student’s digital health attitudes and competencies in 2016, 2021, and 2022. We used *χ*^2^ Calculator by Social Science Statistics to perform the statistical analysis and Effect Size Calculator (effect type: phi) by Statistics Kingdom to calculate effect sizes [50,51]. A *P* value less than .05 was considered statistically significant [52]. Cohen interpretation of the φ is as follows: small effect *φ*=0.1, medium effect *φ*=0.3, and large effect *φ*=0.5 [53]. Based on consultation with a statistician, significance values were corrected for multiple comparisons using the Benjamini-Hochberg procedure to control the false discovery rate (FDR =0.25) [54]. According to the Benjamini-Hochberg procedure, *P* values ≤.03 were found to be statistically significant. The results of data analysis, including the *χ*^2^ statistics (χ²), degrees of freedom (*df*), *P* values, phi coefficients (φ), and Q-values, are reported in Multimedia Appendix 2.

## Results

### Demographic Characteristics of the Study Sample

The sample size of the study included a total of 215 medical students (n=45 in 2016, n=106 in 2021, and n=64 in 2022), with an overall response rate of 53% (43% in 2016, 74% in 2021, and 42% in 2022). No participants were excluded due to incomplete or invalid responses. There were no missing values or other deviations in the questionnaire results. Details regarding gender distribution and age distribution are presented in Table 1.

**Table 1.** Demographic characteristics of the study sample (N=215), including gender distribution and age distribution by decade of birth.

Characteristics	Medical students in 2016 (n=45), n (%)	Medical students in 2021 (n=106), n (%)	Medical students in 2022 (n=64), n (%)
Gender distribution			
Women	27 (60)	56 (53)	34 (53)
Men	18 (40)	50 (47)	27 (42)
Other	—^a^	—	3 (5)
Age distribution (birth decade)			
1960	3 (7)	—	—
1970	3 (7)	—	1 (1.5)
1980	20 (44)	13 (12)	7 (11)
1990	19 (42)	93 (88)	55 (86)
2000	—	—	1 (1.5)

a Not applicable

The average working experience in health care sector was 1.1 (SD 1.3) years in 2021 and 0.9 (SD 1.1) years in 2022. In the 2016 survey, 96% of the students had worked as assistants to medical doctors. The work experience of the students in a field corresponding to their prior education was on average 1.1 (SD 2.5) years in 2021 and 0.9 (SD 2.1) years in 2022. While the 2016 dataset likely includes career changers (persons born in 1960 and 1970) [55], we lack sufficient information about their backgrounds to draw definitive conclusions about this.

### The Usage of Patient-Generated Information and the Role of Applications in Patient Care

**Figure 1.**
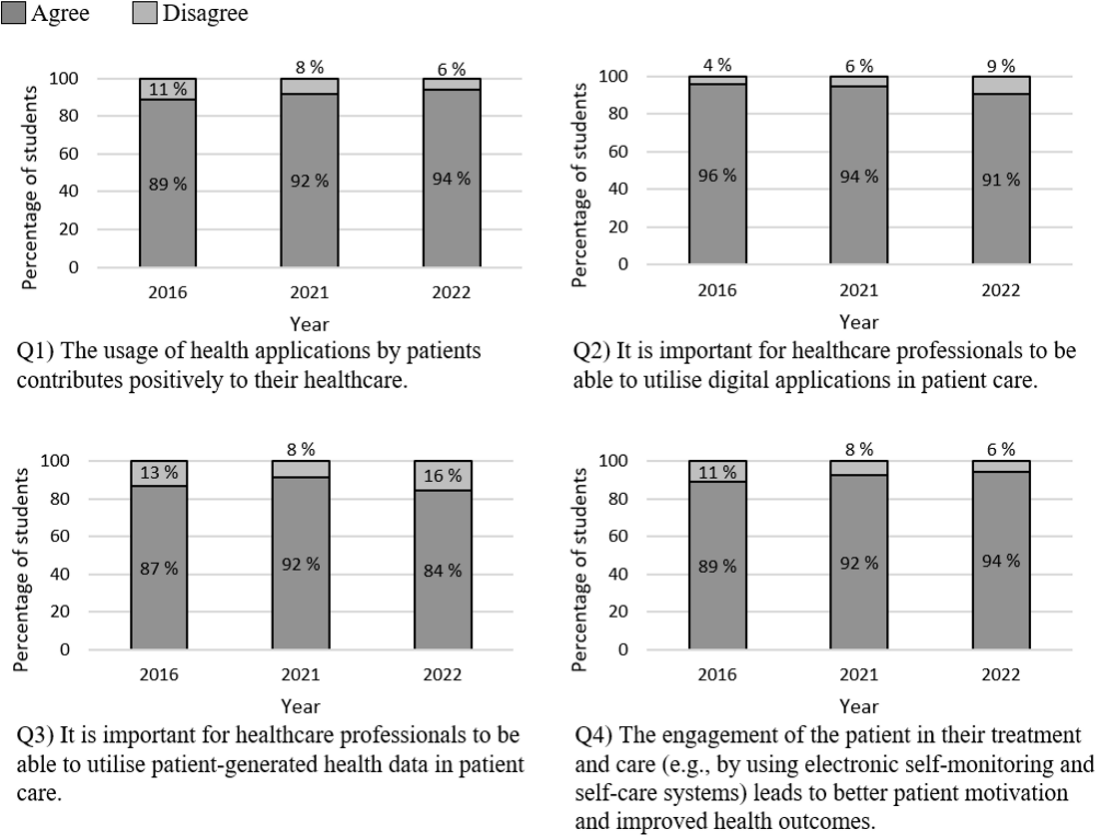
Percentages of students agreeing and disagreeing with each statement related to the theme “The usage of patient-generated information and the role of applications in patient care.” A statistical analysis was performed using a *χ*^2^ test.

Overall, there were no major changes in medical students’ attitudes toward using patient-generated information and the role of digital applications in patient care between 2016, 2021, and 2022 (Figure 1). Students consistently held positive views, asserting that digital applications benefit patients’ health and motivation. They also emphasized the importance of health care professionals being proficient in using these applications.

### Health Information Systems

**Figure 2.**
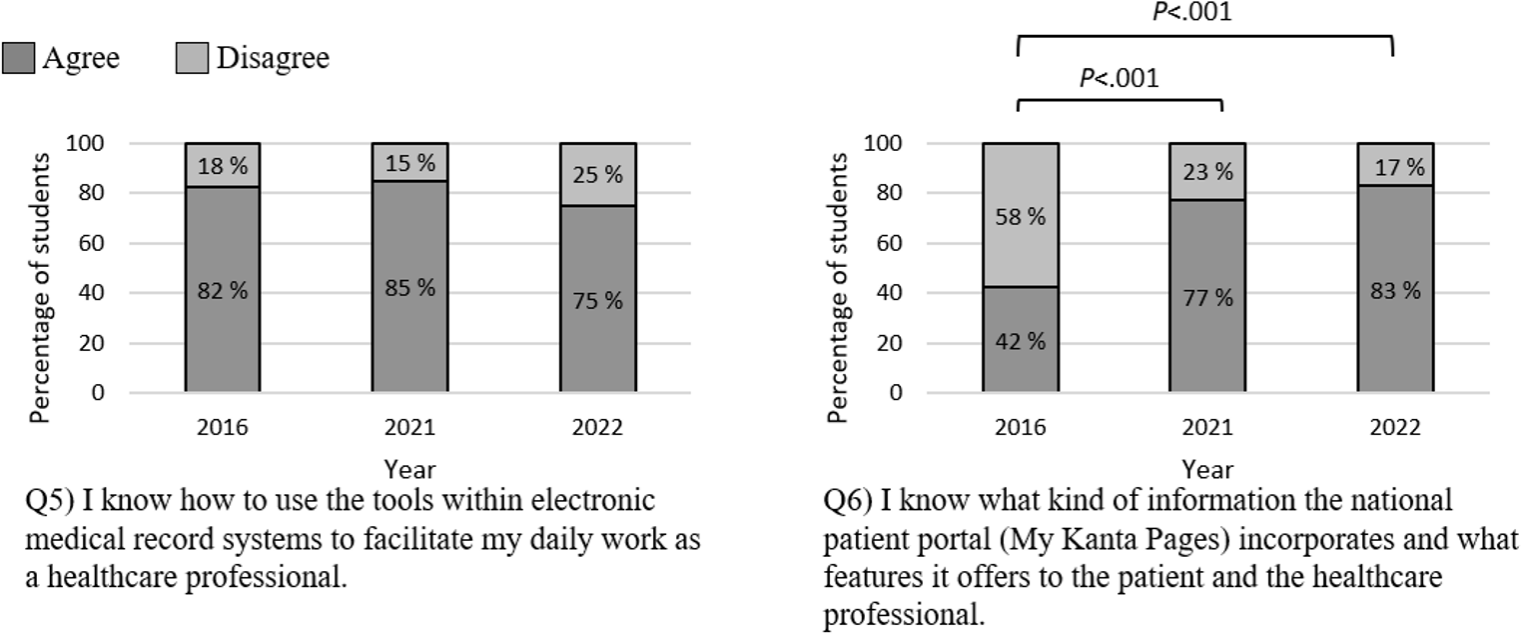
Percentages of students agreeing and disagreeing with each statement related to the theme “Health information systems.” A statistical analysis was performed using a *χ*^2^ test.

There was a significant improvement in the students’ self-perceived knowledge of the information contained in the national patient portal (My Kanta Pages), with agreement increasing by 35 percentage points from 2016 to 2021, and this trend continued in 2022 (Figure 2).

However, there was no significant change in students’ self-perceived skills in using electronic medical records over this period. If anything, the students perceived their skill set to be weaker in 2022 compared to 2016 and 2021, though this difference was not statistically significant.

### Digitalization of the Working Environment

**Figure 3.**
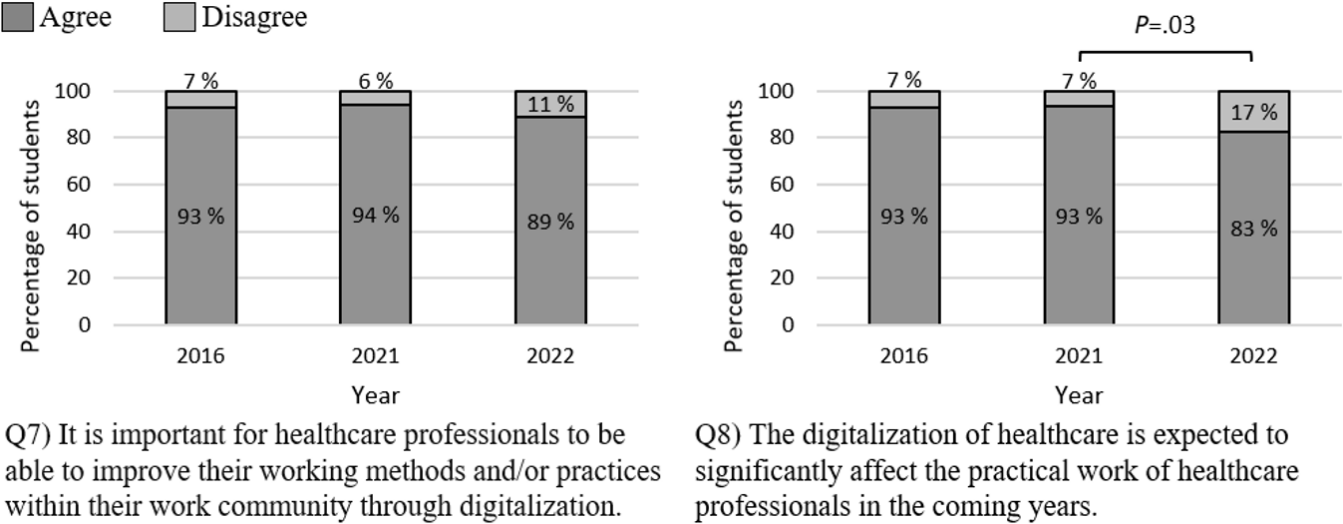
Percentages of students agreeing and disagreeing with each statement related to the theme “Digitalization of the working environment.” A statistical analysis was performed using a *χ*^2^ test.

The survey data indicates that medical students consistently recognize the importance of digitalization in enhancing health care professionals’ working methods (Figure 3). From 2021 to 2022, there was a significant shift in students’ perceptions of how digitalization would affect their working lives in the coming years. In 2021, only 7% of the students disagreed that digitalization significantly changes health care professionals’ practical work, consistent with 2016. By 2022, this disagreement rose to 17%, demonstrating greater uncertainty.

### The Changing Role of Patients and Professionals

**Figure 4.**
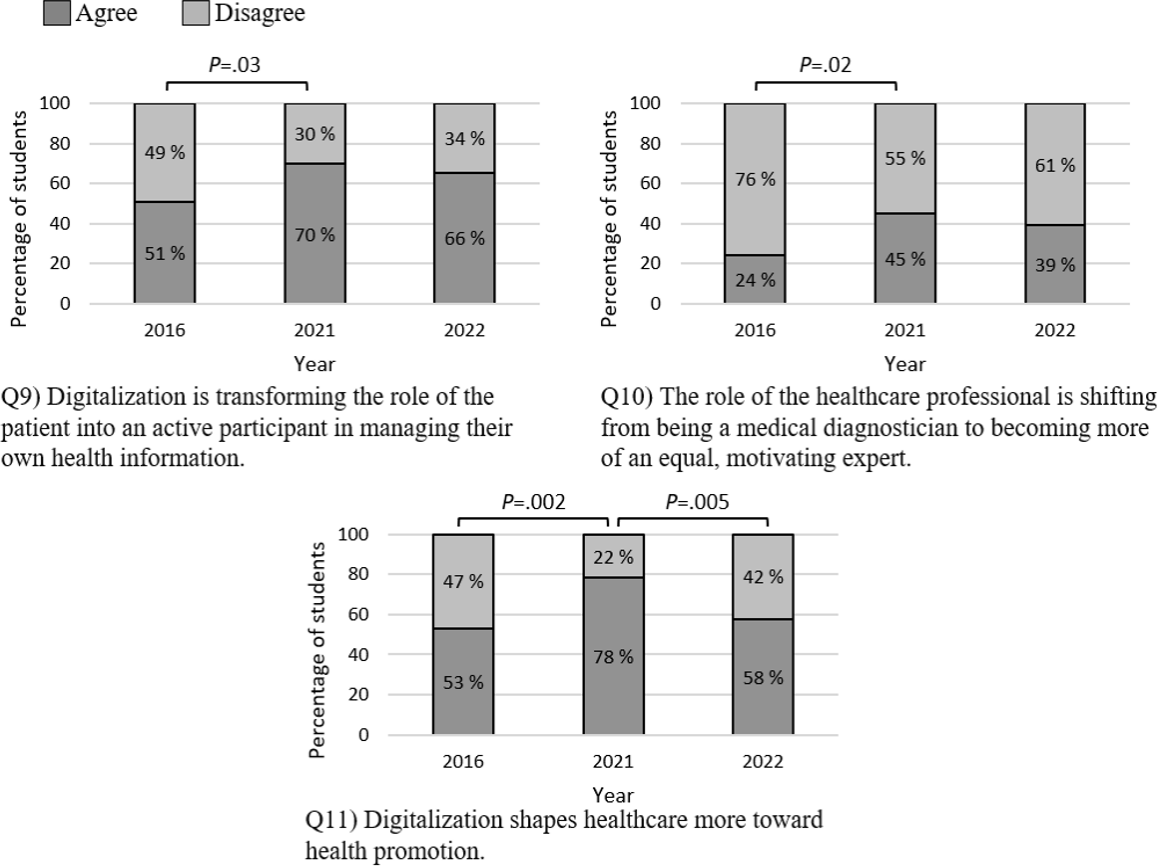
Percentages of students agreeing and disagreeing with each statement related to the theme “The changing role of patients and professionals.” A statistical analysis was performed using a *χ*^2^ test.

Between 2016 and 2021, medical students’ attitudes about the roles of patients and professionals evolved, reflecting increased patient involvement in managing their health information and more equitable relationship between patients and professionals (Figure 4). Additionally, the students’ perceptions of the impact of digitalization on health promotion improved significantly from 2016 to 2021 (with agreement rising from 53% to 78%) but declined notably again by 2022.

### The Culture of Experimentation and Readiness to Participate in Innovation Activities

**Figure 5.**
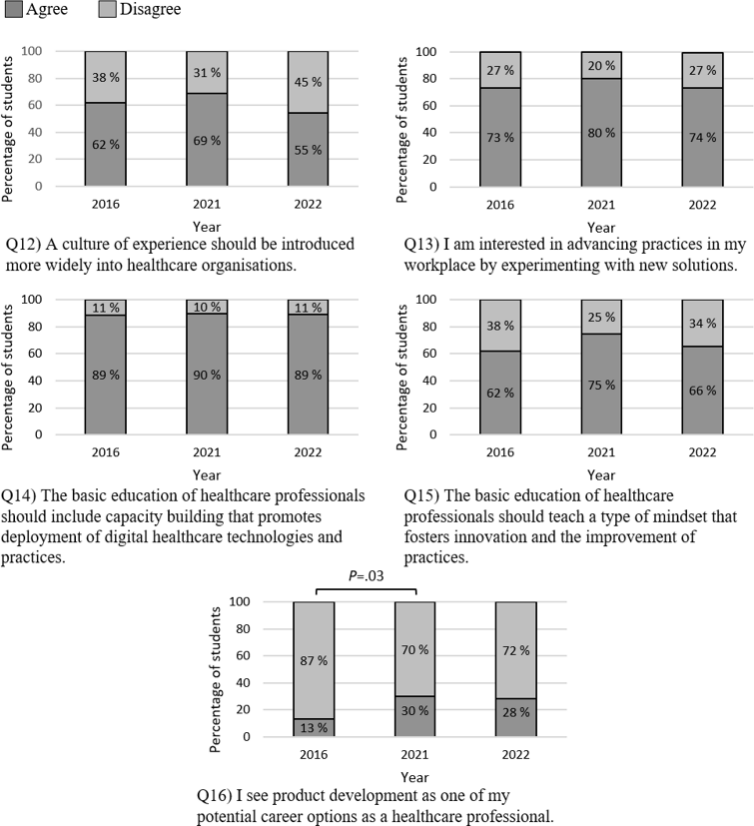
Percentages of students agreeing and disagreeing with each statement related to the theme “The culture of experimentation and readiness to participate in innovation activities.” A statistical analysis was performed using a *χ*^2^ test.

The participating students consistently valued the inclusion of digital health topics in basic medical education across 2016, 2021, and 2022, with no significant changes in attitudes (Figure 5). In addition, alternative career options, such as product development, became increasingly appealing, with the percentage of students considering this path more than doubling from 2016 to 2021 and remaining relatively stable in 2022.

## Discussion

### Principal Findings

Overall, we observed a positive shift in the participating medical students’ attitudes and an improvement in their self-perceived digital competencies between 2016 and 2022. This coincides with advancements in the national health information system, providing an excellent opportunity to assess the outcomes from the CAMM framework viewpoint. Interestingly, we observed that the changes in attitudes were not consistently linear; while there was a positive trend overall, the 2022 results showed signs of stagnation or decline, with the exception of the students’ increasing knowledge of the national patient portal.

To our knowledge, there has been only 1 umbrella review describing health care providers’ attitudes to patient portals using CAMM [56]. This study primarily focused on the adoption of patient portals, revealing predominantly reserved attitudes and concerns among professionals, such as increased workload, insufficient training and resources, accuracy of information, and issues related to patient privacy. Our study contributes to this by extensively mapping the attitudes and digital competencies of future health care professionals, reflecting potential changes connected to the advancements in the national health information system architecture across three different time points. In our work, students’ self-perception of knowledge of the national patient portal had significantly improved. Our data also indicated that, as early as 2016, medical students valued the importance of using patient-generated information and patient engagement via self-care systems relatively high, with 87% and 89% of the students, respectively. This might indicate that while there have been initial concerns among professionals, future professionals are increasingly recognizing the value of digital tools and patient engagement.

In our study, medical students perceived the use of digital applications and patient-generated data in patient care as a positive factor: there were no statistically significant differences in the participating students’ attitudes between 2016, 2021, and 2022 (see Figure 1). From a system adoption point of view, this finding suggests that the widespread use of digital applications had already prompted students to recognize their importance as early as 2016. EU legislation has emphasized the role of medical and health digital applications as a part of patient empowerment and has also recognized potential risk aspects [1,57]. Both the WHO and the European Commission have participated in establishing the European mHealth Knowledge and Innovation Hub as part of the Horizon 2020 project [58]. The initiative aims to support the integration of mHealth services into the national health systems of European countries.

Our results indicate a significant improvement in medical students’ knowledge of the national patient portal since 2016 (see Figure 2, Q6). We believe that this development may be linked to the introduction and implementation of new national health information exchange services in the 2010s and beyond. For example, the implementation of the national patient data repository in public health care in Finland was only completed in late 2015, less than a year before our first data checkpoint [15,19]. Similar nationwide systems for the exchange of health information between patients and professionals can be found in several countries [59], but studies on students’ competencies in the use of these systems are scarce in the literature. Additionally, we discovered that students in 2022 rated their ability to use electronic patient record systems slightly lower than students in both 2021 and 2016. Although this finding was not statistically significant, we know that requirements for recording patient data have become more demanding, and the functionalities of the systems have increased [60]. These trends may have influenced the students’ perceptions in 2022.

Prior research indicates that medical students have concerns regarding the impact of digitalization on the patient-doctor relationship [28,35]. Our study reveals a shift in attitudes related to the roles of patients and professionals. We discovered a statistically significant difference between 2016 and 2021 in the attitudes of participating students toward patients’ roles in managing their health information and collaborating with professionals. Additionally, students in 2021 recognized the role of digitalization in shaping health care toward health promotion. These findings align with both national and EU health strategies, which emphasize electronic services to support the active role of citizens in maintaining their own well-being [1,61]. In Finland, the national health exchange services were complemented by a personal health record repository service (Kanta Personal Health Record or Kanta PHR), which entered its first-phase production in 2018 [19]. The service allows citizens to input, store, and share their well-being data with professionals. At the EU level, the EU is establishing a European Health Data Space ecosystem aiming to empower individuals of member states with control over their health data [9].

Overall, we saw slightly more reserved attitudes toward digital health in 2022 compared to 2021. The assertion to presentation ‘The digitalization of healthcare is expected to significantly affect the practical work of healthcare professionals in the coming years’ was notably less supported in 2022 compared to 2021 (see Figure 3, Q8). This trend suggests that electronic health services and tools have become fully integrated into the health care system. It may even indicate that, from the students’ perspective, digitalization has reached its apex in health care. This shift could be linked to the advancements in digital health education in the basic medical training in Finland, such as the completion of the national MEDigi project in 2021 and the introduction of its eHealth competence areas in 2020 [43,44].

Another noteworthy discovery was the significant increase in the number of students who agreed that “Digitalization shapes health care more toward health promotion” between 2021 and 2022, followed by a notable decrease in agreement between 2021 and 2022 (see Figure 4, Q11). This change in attitudes could be partly linked to the “care debt” and prolonged waiting times for treatment that emerged during the global health emergency caused by the COVID-19 pandemic in 2020‐2023 [62,63]. It is also possible that after over 2 years of remote and blended education, students in 2022 may be exhibiting signs of digital fatigue [64,65]. As a result, the enthusiasm for digitalization driven by the COVID-19 pandemic may have started to decline by 2022. Nevertheless, these trends require further research to fully comprehend the underlying causes behind the phenomenon. The more reserved attitudes of the participating students and their somewhat lower self-assessed skills suggest the necessity for an increased focus on digital training for future health care professionals. This is crucial to ensure their competencies align with the broad health strategies in Europe and on a global scale.

The medical students who participated in this study in 2016, 2021, and 2022 were aligned in their belief that the basic education of health care professionals should include capacity building promoting the deployment of digital health solutions (see Figure 5, Q14). This consensus resonates with findings from previous studies [26-34]. The students’ attitudes shifted positively toward alternative career paths such as product development (see Figure 5, Q16). This change was statistically significant between 2016 and 2021: in 2016, only 13% of students agreed with this claim, whereas almost a third of the students agreed with it in 2021. Although the difference between 2016 and 2022 was not statistically significant, the trend was consistent with percentages of 13% and 28%, respectively. This is an important finding, as end-user involvement is considered a critical success factor in information technology projects [66-69]. A Finnish study revealed that younger physicians were more eager to participate in health information system development compared to their older counterparts [70]. Additionally, research indicates that interdisciplinary collaborations between health care and engineering professionals can foster innovation and new practices, underscoring the importance of interprofessional education [71,72]. However, introducing digital health topics and innovation activities into medical curricula has proven to be challenging in practice due to crowded curricula designs and competing interests [29,73-75].

### Strengths and Limitations

Our overall response rate was 53%. Our study sample was collected from one of the 5 Finnish medical universities at 3 different time points. The results are likely to be applicable to other Finnish medical faculties given the similar surrounding health care system and relatively homogeneous education system [20,43]. Furthermore, these findings may also have relevance to other countries, particularly those with similar health care systems, health care information system architecture, and medical curriculum design. However, confirming this would necessitate further research.

There are also several limitations to this study. Firstly, we had a relatively restricted sample size from 1 education institution, which may affect the generalizability of the findings. While cross-sectional studies are useful for examining associations, causal relationships cannot be established, which should be considered when interpreting the findings. We relied on self-assessment to evaluate competencies in this study, which means we were unable to measure absolute changes in skills and knowledge. Because of minor variations in the collection of demographic characteristics among the study sample, a statistical comparison of the students’ demographic characteristics could not be conducted. Furthermore, the dichotomization of variables may potentially hinder the reflection of results in real-world situations.

Future research should aim to include larger and more diverse samples from multiple institutions and possibly from other health care professions to enhance the generalizability of the findings. Additionally, longitudinal studies could provide more insight into causal relationships and how the attitudes and capabilities of the students may shift after entering working life. More information on the best teaching strategies for digital health topics is needed to facilitate optimal learning outcomes for health care professionals.

### Conclusions

There has been a shift in self-perceived digital competence and attitudes of medical students over the years, potentially influenced by the development of national health information system architecture. However, this change has not followed a completely linear trajectory, and students’ attitudes toward digital health have become somewhat more reserved in certain areas over time, particularly regarding the extent to which digitalization alters health care processes and its role in health promotion.

To address these gaps, medical educational institutions and policy makers should consider integrating more digital health topics into curricula, offer practical experience with digital health technologies, and implement effective teaching strategies for digital health. Given the rapid evolutions of the health care field, it is crucial to ensure the professionals keep up with the changes of this dynamic working environment. The integration of digital health education should be carefully considered and evaluated to ensure it meets the needs of both students and health care systems.

## Supplementary material

10.2196/67423 Multimedia Appendix 1 An English translation of the study questionnaire.

10.2196/67423 Multimedia Appendix 2 The results of the data analysis detailing the chi-square statistics (χ²), degrees of freedom (df), P-values, phi coefficients (φ), and Q-values.

## Abbreviations

CAMM: clinical adoption meta-model
EU: European Union
WHO: World Health Organization
