# Supplementary material for: The Evolution of Medical Student Competencies and Attitudes in Digital Health Between 2016 and 2022: Comparative Cross-Sectional Study
Source: JMIR Med Educ. 2025 Jul 31;11:e67423. doi: 10.2196/67423 (PMC12313084; doi:10.2196/67423)
Supplement: Multimedia Appendix 1 [file mededu-v11-e67423-s001.pdf]

Multimedia Appendix 1. An English translation of the study questionnaire

The usage of patient-generated information and the role of digital applications in patient care

1) The usage of health applications by patients contributes positively to their healthcare.
2) It is important for healthcare professionals to be able to utilize digital applications in patient care.
3) It is important for healthcare professionals to be able to utilize patient-generated health data in patient care.
4) The engagement of the patient in their treatment and care (e.g., by using electronic self-monitoring and self-care systems) leads to a better patient motivation and improved health outcomes.

Health information systems

5) I know how to use the tools within electronic medical record systems to facilitate my daily work as a healthcare professional.
6) I know what kind of information the national patient portal (My Kanta Pages) incorporates and what features it offers to the patient and the healthcare professional.

Digitalization of the working environment

7) It is important for healthcare professionals to be able to improve their working methods and/or practices within their work community through digitalization.
8) The digitalization of healthcare is expected to significantly affect the practical work of healthcare professionals in the coming years.

The changing role of patients and professionals

9) Digitalization is transforming the role of the patient into an active participant in managing their own health information.
10) The role of healthcare professional is shifting from being a medical diagnostician to becoming more of an equal, motivating expert.
11) Digitalization shapes healthcare more toward health promotion.

The culture of experimentation and readiness to participate in innovation activities.

12) A culture of experimentation should be introduced more widely into healthcare organizations.

13) I am interested in advancing practices in my workplace by experimenting with new solutions.

14) The basic education of healthcare professionals should include capacity building that promotes deployment of digital healthcare technologies and practices.

15) The basic education of healthcare professionals should teach a type of mindset that fosters innovation and the improvement of practices.

16) I see product development as one of my potential career options as a healthcare professional.
