# Supplementary material for: The Evolution of Medical Student Competencies and Attitudes in Digital Health Between 2016 and 2022: Comparative Cross-Sectional Study
Source: JMIR Med Educ. 2025 Jul 31;11:e67423. doi: 10.2196/67423 (PMC12313084; doi:10.2196/67423)
Supplement: Multimedia Appendix 2 [file mededu-v11-e67423-s002.pdf]

**Multimedia Appendix 2.** The results of the data analysis detailing the chi-square statistics ($\chi^2$), degrees of freedom (df), P-values, phi coefficients ($\phi$), and Q-values.

| Statement | 2016 vs. 2021 ($\chi^2$, df, P-value, $\phi$, Q-value) | 2021 vs. 2022 ($\chi^2$, df, P-value, $\phi$, Q-value) | 2016 vs. 2022 ($\chi^2$, df, P-value, $\phi$, Q-value) |
|---|---|---|---|
| Q1 | 0.510, 1, 0.475, 0.058, 0.167 | 0.102, 1, 0.749, 0.025, 0.203 | 0.824, 1, 0.364, 0.087, 0.125 |
| Q2 | 0.093, 1, 0.760, 0.025, 0.214 | 0.839, 1, 0.360, 0.070, 0.115 | 0.945, 1, 0.331, 0.093, 0.104 |
| Q3 | 0.828, 1, 0.363, 0.074, 0.120 | 2.046, 1, 0.153, 0.110, 0.083 | 0.111, 1, 0.739, 0.032, 0.198 |
| Q4 | 0.510, 1, 0.475, 0.058, 0.172 | 0.102, 1, 0.749, 0.025, 0.208 | 0.824, 1, 0.364, 0.087, 0.130 |
| Q5 | 0.170, 1, 0.680, 0.034, 0.188 | 2.563, 1, 0.109, 0.123, 0.063 | 0.803, 1, 0.370, 0.086, 0.135 |
| Q6 | 17.608, 1, <0.001, 0.342, 0.010 | 0.726, 1, 0.394, 0.065, 0.141 | 19.415, 1, <0.001, 0.422, 0.005 |
| Q7 | 0.057, 1, 0.811, 0.019, 0.224 | 1.574, 1, 0.210, 0.096, 0.089 | 0.578, 1, 0.447, 0.073, 0.162 |
| Q8 | 0.000, 1, 0.989, 0.001, 0.245 | 4.722, 1, 0.030, 0.167, 0.042 | 2.613, 1, 0.106, 0.155, 0.057 |
| Q9 | 4.809, 1, 0.028, 0.179, 0.031 | 0.323, 1, 0.570, 0.044, 0.177 | 2.312, 1, 0.128, 0.146, 0.078 |
| Q10 | 5.762, 1, 0.016, 0.195, 0.026 | 0.630, 1, 0.427, 0.044, 0.146 | 2.553, 1, 0.110, 0.153, 0.068 |
| Q11 | 9.538, 1, 0.002, 0.251, 0.016 | 8.069, 1, 0.005, 0.218, 0.021 | 0.215, 1, 0.643, 0.044, 0.182 |
| Q12 | 0.630, 1, 0.427, 0.065, 0.151 | 3.463, 1, 0.063, 0.143, 0.047 | 0.615, 1, 0.433, 0.075, 0.156 |
| Q13 | 0.869, 1, 0.351, 0.076, 0.109 | 1.048, 1, 0.306, 0.079, 0.099 | 0.000, 1, 0.990, 0.001, 0.250 |
| Q14 | 0.018, 1, 0.893, 0.011, 0.229 | 0.013, 1, 0.908, 0.009, 0.234 | 0.001, 1, 0.977, 0.003, 0.240 |
| Q15 | 2.317, 1, 0.128, 0.124, 0.073 | 1.542, 1, 0.214, 0.101, 0.094 | 0.133, 1, 0.715, 0.035, 0.193 |
| Q16 | 4.766, 1, 0.029, 0.178, 0.037 | 0.082, 1, 0.775, 0.022, 0.219 | 3.367, 1, 0.067, 0.176, 0.052 |

| | | | |
|---|---|---|---|
| Q1 | 0.5100, 1, .475, 0.0581, 0.1667 | 0.1024, 1, .749, 0.0245, 0.2031 | 0.8242, 1, .364, 0.0870, 0.1250 |
| Q2 | 0.0931, 1, .760, 0.0248, 0.2135 | 0.8393, 1, .360, 0.0703, 0.1146 | 0.9445, 1, .331, 0.0931, 0.1042 |
| Q3 | 0.8280, 1, .363, 0.0741, 0.1198 | 2.0461, 1, .153, 0.1097, 0.0833 | 0.1108, 1, .739, 0.0319, 0.1979 |
| Q4 | 0.5100, 1, .475, 0.0581, 0.1719 | 0.1024, 1, .749, 0.0245, 0.2083 | 0.8242, 1, .364, 0.0870, 0.1302 |
| Q5 | 0.1702, 1, .680, 0.0336, 0.1875 | 2.5626, 1, .109, 0.1228, 0.0625 | 0.8027, 1, .370, 0.0858, 0.1354 |
| Q6 | 17.6082, 1, .000, 0.3415, | 0.7260, 1, .394, 0.0654, 0.1406 | 19.4146, 1, .000, 0.4220, |
| Q7 | 0.0571, 1, .811, 0.0195, 0.2240 | 1.5736, 1, .210, 0.0962, 0.0885 | 0.5784, 1, .447, 0.0729, 0.1615 |
| Q8 | 0.0002, 1, .989, 0.0012, 0.2448 | 4.7217, 1, .030, 0.1667, 0.0417 | 2.6126, 1, .106, 0.1548, 0.0573 |
| Q9 | 4.8086, 1, .028, 0.1785, 0.0312 | 0.3227, 1, .570, 0.0436, 0.1771 | 2.3122, 1, .128, 0.1456, 0.0781 |
| Q10 | 5.7622, 1, .016, 0.1953, 0.0260 | 0.6302, 1, .427, 0.0436, 0.1458 | 2.5525, 1, .110, 0.1530, 0.0677 |
| Q11 | 9.5377, 1, .002, 0.2513, 0.0156 | 8.0694, 1, .005, 0.2179, 0.0208 | 0.2151, 1, .643, 0.0444, 0.1823 |
| Q12 | 0.6299, 1, .427, 0.0646, 0.1510 | 3.4634, 1, .063, 0.1427, 0.0469 | 0.6150, 1, .433, 0.0751, 0.1562 |
| Q13 | 0.8693, 1, .351, 0.0759, 0.1094 | 1.0479, 1, .306, 0.0785, 0.0990 | 0.0001, 1, .990, 0.0010, 0.2500 |
| Q14 | 0.0180, 1, .893, 0.0109, 0.2292 | 0.0132, 1, .908, 0.0088, 0.2344 | 0.0008, 1, .977, 0.0027, 0.2396 |
| Q15 | 2.3169, 1, .128, 0.1239, 0.0729 | 1.5419, 1, .214, 0.1011, 0.0938 | 0.1331, 1, .715, 0.0349, 0.1927 |
| Q16 | 4.7655, 1, .029, 0.1777, 0.0365 | 0.0819, 1, .775, 0.0219, 0.2188 | 3.3668, 1, .067, 0.1758, 0.0521 |
